# Intravitreal safety profiles of sol-gel mesoporous silica microparticles and the degradation product (Si(OH)_4_)

**DOI:** 10.1080/10717544.2020.1760401

**Published:** 2020-05-12

**Authors:** Yaoyao Sun, Kristyn Huffman, William R. Freeman, Michael J. Sailor, Lingyun Cheng

**Affiliations:** a Department of Ophthalmology, Jacobs Retina Center at Shiley Eye Institute, University of California, San Diego, CA, USA;; b Department of Chemistry and Biochemistry, University of California, San Diego, CA, USA

**Keywords:** Intravitreal drug delivery, sol-gel mesoporous silica, silicic acid cytotoxicity, silica pore size and ocular toxicity, rabbit and guinea pig eyes

## Abstract

Mesoporous silica has attracted significant attention in the drug delivery area; however, impurities can be a source of toxicity. The current study used commercial microparticles produced at large scale in a well-controlled environment. Micrometer sized mesoporous silica particles were acquired through a commercial vendor and pore structures were characterized by SEM. The three silica particle formulations had a diameter of 15 micrometers and three different pore sizes of 10 nm, 30 nm, and 100 nm. The fourth formulation had particle size of 20–40 micrometers with 50 nm pores. Before *in vivo* tests, an in vitro cytotoxicity test was conducted with silicic acid, derived from the sol-gel particles, on EA.hy926 cells. Low concentration (2.5 µg/mL) of silicic acid showed no cytotoxicity; however, high concentration (25 µg/mL) was cytotoxic. *In vivo* intravitreal injection demonstrated that 15 um silica particles with 10 nm pore were safe in both rabbit and guinea pig eyes and the particles lasted in the vitreous for longer than two months. Formulations of with larger pores demonstrated variable localized vitreous cloudiness around the sol-gel particle depot and mild inflammatory cells in the aqueous humor. The incidence of reaction trended higher with larger pores (10 nm: 0%, 30 nm: 29%, 50 nm: 71%, 100 nm: 100%, *p* < .0001, Cochran Armitage Trend Test). Sol-gel mesoporous silica particles have uniform particle sizes and well-defined pores, which is an advantage for implantation via a fine needle. Selected formulations may be used as an intraocular drug delivery system with proper loading and encapsulation.

## Introduction

In recent years, various forms of porous silicon (pSi) have been used as vehicles for drug delivery (Anglin et al., 2008; Salonen et al., 2008). For example, mesoporous silica nanoparticles have been extensively explored for drug delivery in general (Siafaka et al., 2016, 2019). Compared with traditional drug delivery vehicles, such as liposomes or polymer particulates, pSi offers tunable nano-scaled pores for variously-sized payloads and adjustable drug release rates (Hou et al., 2014). In addition, pSi can be modified using various surface chemistries to alter its degradation half-life (Cheng et al., 2008) and its degradation products are water-miscible for clean elimination (Nieto et al., 2013). These properties make pSi very attractive for intravitreal drug delivery (Nan et al., 2014). Generally, functionalized pSi has demonstrated good biocompatibility both in vitro (Alvarez et al., 2009) and in vivo (Bimbo et al., 2010). However, the eye is a special organ that demands clear media for clear vision and the sensory retina is completely exposed to pSi particles after intravitreal injection. Differing from other organs, the eye is minimally tolerable to adverse reactions or inflammation associated with the injection of foreign material into the vitreous. Some studies have reported concentration-dependent pSi cytotoxicity on retinal pigment epithelium cells in vitro (Korhonen et al., 2016). It is known that porous silicon is biodegradable into silicic acid (Si[OH_4_]), a material that is also naturally present in human tissues (Reffitt et al., 1999). Even so, the safety profile of silicic acid on ocular cells is scarce in the literature. Porous silicon is usually employed as a long-term drug delivery system, which means the retina and anterior segment will be constantly exposed to various concentrations of silicic acid because a large portion of the degradation product is eliminated through the anterior chamber of the eye globe (Nieto et al., 2013). In an in vivo study, inflammatory reactions were noted when a piece of pSi was implanted under the conjunctiva (Low et al., 2009). From our experience, we have also occasionally noted variable vitreous reactions following the intravitreal injection of empty pSiO_2_ particles prepared from electrochemical etching in the lab (Nieto et al., 2013). Interestingly, these occasional mild reactions do not occur with drug-loaded particles (Chhablani et al., 2013; Hartmann et al., 2013; Nan et al., 2014). It is possible that the loaded drug suppresses the reaction or that the drug loading process changes the surface properties of the pSiO_2_. The pSi particles reported in literature are fabricated from electrochemical etching using hydrofluoric acid in academic labs. Impurities may have been introduced during the etching, particle production, or surface functionalization, which may be responsible for the variable reactions observed.

In contrast to electrochemical etching of silicon substrate and subsequent oxidation in the research lab, mesoporous silica particles synthetized by the sol-gel process have been used for drug delivery in various non-ophthalmic applications (Owens et al., 2016; Vlasenkova et al., 2019). The sol-gel process in a large-scale production has many advantages, including significantly higher purity and uniform particle size and pore size. In the current study, sol-gel silica particles without payload are used to generate soluble silicic acid to test cytotoxicity in vitro and sol-gel silica articles with various pore sizes were evaluated in vivo after intravitreal injection. Eye specific information about sol-gel silica particles is meager in literature and the current study aims to explore intravitreal safety of mesoporous sol-gel silica microparticles in the context of a drug delivery vehicle.

## Materials and methods

### Materials

Sol-gel particles were purchased from Silicycle (Quebec City, Canada https://www.silicycle.com/products/siliasphere). Four different formulations of sol-gel particles were acquired. A chromogenic Limulus Amebocyte Lysate (LAL) endotoxin assay was performed by American Testing Lab (San Diego, CA) on these sol-gel silica particles before use in the studies. The human endothelial cell line, EA.hy926, was purchased from American Type Culture Collection (CRL-2922) and grown in Dulbecco’s modified Eagle’s medium (DMEM; Gibco), supplemented with 10% fetal bovine serum (FBS; Corning) and 1% Penicillin-Streptomycin (5,000 U/mL, Gibco). Silicic acid, 80 mesh was purchased from Sigma.

### Study design and methods

#### Cell culture and cytotoxicity

EA.hy926 cells, a hybrid of a human umbilical vein endothelial cell and a lung carcinoma cell, were used in order to investigate the cytotoxicity of silicic acid. We are concerned with the integrity of the retina-blood barrier, which consists of endothelial cells and the tight junctions between them. EA.hy926 is a permanent human cell line that preserves many features of human vein endothelial cells. This cell line replicates faster than primary endothelial cells, which provides uniformity and a margin of safety for toxicity testing. The purchased sol-gel particles were used to generate the silicic acid to be tested on the cell line while commercial chemical grade silicic acid was used as the control. 10 mg of sol-gel particles (15 µm particle diameter/10 nm pore diameter) were dissolved in 10 mL of 50 mM sodium hydroxide and stirred at 37 °C for 1 week to generate the saturated silicic acid solution. 10 mg of purchased silicic acid (Sigma-Aldrich Corp., St. Louis, MO) was dissolved accordingly and served as a control. One milliliter of the supernatant was harvested and its pH adjusted to 7.0 by 1 M HCl. The silicic acid solutions were filtered with a 0.22 mm filter for sterilization. The concentration of silicic acid was determined by inductively coupled plasma-optical emission spectroscopy (ICP-OES) in an argon plasma spectrometer (Optima 3000 DV; Perkin Elmer, Norwalk, CT) equipped with a standard torch, Scott-type spray chamber, GemTip cross-flow nebulizer and an AS-90 auto sampler (Perkin Elmer, Norwalk, CT) (Nieto et al., 2013). The silicic acid solutions were diluted as needed in phosphate buffered saline (PBS) according to the concentrations determined by ICP-OES.

The EA.hy926 cells were cultured with DMEM containing 10% (by volume) FBS and 1% Penicillin-Streptomycin and were incubated at 37 °C 5% CO2. After 24 hours to allow the cells to attach, the culture medium was replaced with a fresh mixture of 1-part silicic acid solution and 4 parts culture media. The final concentrations of silicic acid in medium were 25 µg and 2.5 ug/mL. Cells grown in 1-part PBS and 4-parts culture media were used as control. The cytotoxicity assay (WST-1, Roche Diagnostics Corp., Indianapolis, IN) was performed after 5 days and 5 weeks of exposure to silicic acid per the manufacturer’s instructions and our lab’s previously published work (Kim et al., 2012). Silicic acid solution, or PBS as control, was added to the medium at each medium change and passage. At 5 days and 5 weeks, cells were seeded onto a 96-well plate at a density of 10,000 cells/well and allowed to attach for 24 hours. After attachment, 10 μL of the WST-1 reagent was added to each well and the plate was incubated at 37 °C and 5% CO2 for 2 hours. The optical density of the developed color was measured at 440 nm.

#### In vitro silicic acid release from sol-gel pSi particles

The pore size of an intravitreal particulate drug delivery system is an important parameter in terms of release rate of the payload and the elimination rate of the vehicle material. Characterizing the dissolution rate and vitreous elimination profile of silicic acid is an important part of optimizing the intravitreal delivery system using mesoporous silica particles. In order to investigate the rate at which sol-gel silica degrades into silicic acid, an in vitro release experiment was carried out. We tested 3 different sized particles for in vitro silicic acid release, 15 µm/10 nm, 25–45 µm/50 nm and 15 µm/100 nm (particle diameter/pore diameter). Briefly, 2 mg of sol-gel silica particles was weighed into a 1.5 mL microcentrifuge tube with 1200 µL of PBS. The vials were incubated at 37 °C. The vials were centrifuged at 5,000 rpm for 5 minutes, and 1000 uL of the supernatant was collected and stored at –80 °C. Then, 1000 uL of PBS was added back to each of the tubes to restore the volume of the dissolution medium. The experiment was carried out daily and all samples were analyzed by the end of week 3. The concentration of silicic acid was determined by ICP-OES.

#### In vivo ocular toxicity study

Two animal species were used for the ocular toxicity studies. Rabbit eye is an inexpensive model with a relatively close amount of vitreous to the human eye when compared to rodents. Vitreous volume is an important parameter for the development of an intravitreal drug delivery system. In addition, guinea pig eyes were also used as a second species for the confirmatory study. According to our published data, a large percentage of the vitreal silicic acid is eliminated through anterior chamber circulation (Nieto et al., 2013) making the guinea pig eye a good model as it is more sensitive to drug toxicity, especially ciliary toxicity and intraocular pressure changes (Taskintuna et al., 1997). Twenty-six New Zealand Pigmented rabbits were used to study the safety and stability of the sol-gel silica particles after the intravitreal injection. Ten Guinea pigs were used for the confirmatory ocular toxicity study. All animal handling was carried out in adherence to the ARVO Statement for the Use of Animals in Ophthalmic and Vision Research. Only one eye of each animal was injected with the porous silicon particles, and the contralateral eye was injected with the same volume of sterile saline to serve as the control. A 27-gauge needle was used to deliver the suspension into the vitreous through the pars plana under the direct view of a surgical microscope. Mass balance was used to quantify the injected pSi amount per eye.

After intravitreal injection, the eyes were monitored with an indirect ophthalmoscope, tonometer and biomicroscopic slit-lamp on day 3, day 7 and weekly thereafter (Cheng et al., 1999; 2004). Inflammatory cells in aqueous humor were graded using Standardization of Uveitis Nomenclature criteria (Jabs et al., 2005). For localized vitreous haze around the silica depot, gradings were performed as follows: 0: clear vitreous and particles; 1+: hazy particles and hazy vitreous surrounding; 2+: silica is cloudy or opaque and/or membranes in vitreous nearby; 3+: silica is a white mass along with whitish vitreous surrounding; 4+: silica is a white mass with nearby organized vitreous membrane and tractional retinal detachment. Fundus photography was carried out at each observational time point. As previously described, electroretinograms (ERG) were recorded from all eyes of the animals prior to euthanasia, either at week 4 (short term) or week 8 (long term) (Cheng et al., 2002). Fundus fluorescein angiography (FA) and optical coherence topography (OCT) were also carried out at the study end-point.

After euthanasia, the eye globes were enucleated and fixed in 10% formalin. Terminal deoxynucleotidyl transferase dUTP nick end labeling (TUNEL) and hematoxylin and eosin (H&E) staining were performed on paraffin-embedded sections. DNA strand breaks were labeled and detected by the TUNEL method using the Apotag kit (MilliporeSigma, Burlington, MA) according to the manufacturer’s protocol. Study controls were made from sections of the contralateral eye. The positive control was made by incubating the slides in 1x DNase buffer (Promega, Madison, WI) for 5 minutes at room temperature before the assay as we have previously shown (Wang et al., 2011). The negative control was created by omitting the terminal deoxynucleotidyl transferase incubation step.

### Statistical analysis

OD values form the cytotoxicity assay were compared among the differently sourced silicic acid as well as different concentration levels with a nested regression model (concentration nested in the different source groups). For the in vivo study, IOP and ERG readings were recorded multiple times from the same eyes. Pooled IOP and ERG parameters were analyzed using a t-paired test between the right and left eyes. The left eyes received the equivalent volume of BSS as a control. All analyses were performed using JMP^®^ Pro 14.3.0. *p*-values smaller than .05 were considered to be significant.

## Results

### Characteristics of the sol-gel silica particles

The sol-gel particles utilized in this study and their parameters are shown in Table 1. The four formulations consist of different combinations of particle size and pore size. Endotoxin levels in all formulations were below the detection limit (0.1 EU/mL) of the LAL assay. There are two particle sizes: 15 µm and 25–40 µm. These particle sizes were selected for easy passage through a 27-gauge needle which has a 210 µm inner diameter. There are four pore sizes: 10 nm, 30 nm, 50 nm, and 100 nm. The pore sizes were selected according to payload types. The 10 nm pore is useful for loading most small molecule drugs while the pores greater than 30 nm would be suitable for loading small peptides up to larger protein therapeutics.

**Table 1.** Parameters of the sol-gel silica particles.

Particle types	1	2	3	4
Particle size (µm)	15	15	25–40	15
Pore diameter (nm)	10	30	50	100
Surface area (m^2^/g)	390	108	48	19
Pore Volume (mL/g)	1.02	0.83	0.67	0.8

**Figure 1.**
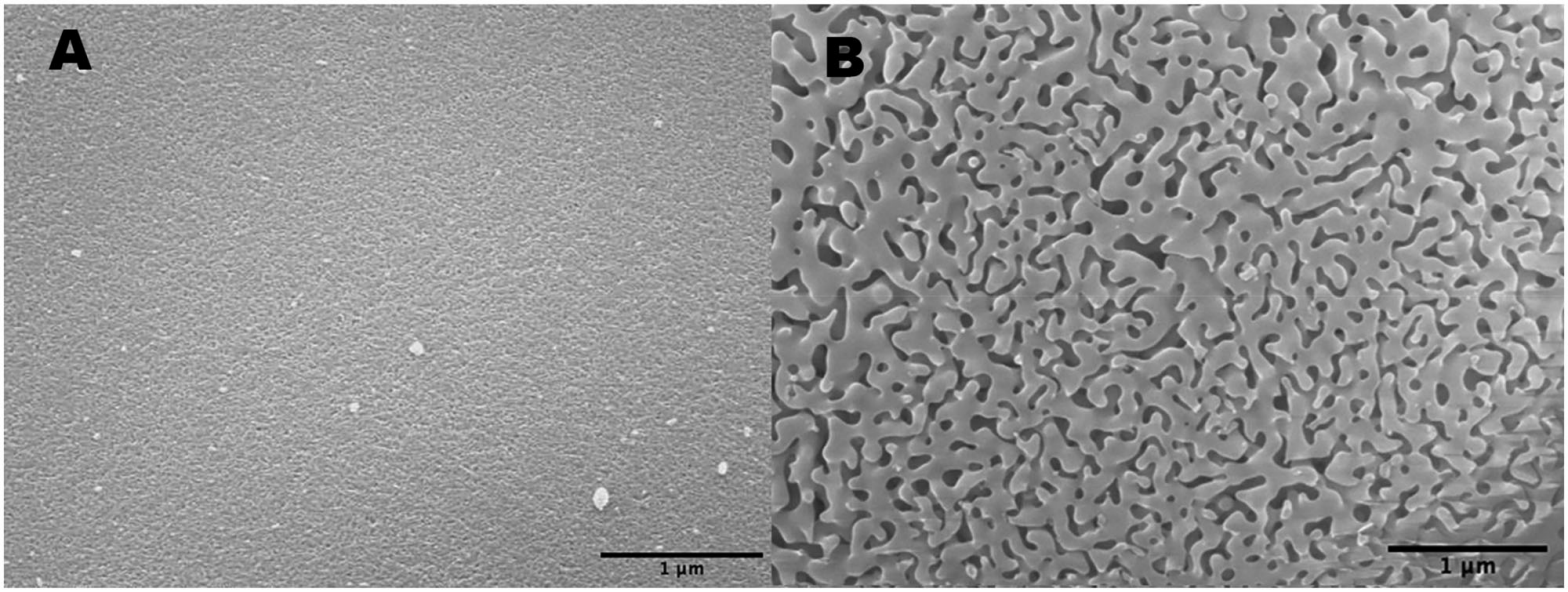
SEM images of the sol-gel silica pores: (A) pore = 10 nm; (B) pore = 100 nm.

The scanning electron microscope (SEM) images of pSi particles with different pore sizes are demonstrated in Figure 1.

### Cytotoxicity of silicic acid

**Figure 2.**
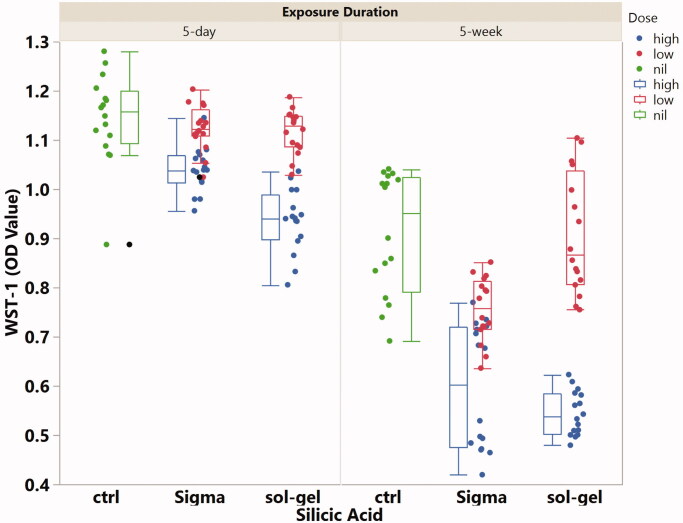
The boxplots of the OD values from the WST-1 cell viability assay stratified by the source of silicic acid, concentration of silicic acid, and exposure time of silicic acid to the cultured EA.hy926 cells.

WST-1 cell viability data was analyzed by the exposure time because senescence is a known factor affecting the health of cultured cells. Under 5 days of silicic acid exposure, low concentration (2.5 µg/mL) of Sigma and sol-gel sourced silicic acid showed no cytotoxicity when compared with the concurrent control (Figure 2); however, high concentration (25 µg/mL) from both sources was cytotoxic (least square mean, LSM, of OD value 1.14 for the control, 1.04 for Sigma silicic acid, 0.94 for sol-gel sourced silicic acid, *p* < .0001). Under 5 weeks of silicic acid exposure, low concentration sol-gel silicic acid was not cytotoxic while low concentration Sigma was cytotoxic when compared with the concurrent control (Figure 2) (LSM of OD value 0.91 for the control, 0.91 for sol-gel sourced silicic acid, 0.76 for Sigma silicic acid, *p* < .0001). In addition, both sources of high concentration silicic acid were cytotoxic (LSM = 0.60 for Sigma sourced and LSM = 0.54 for sol-gel sourced).

### *In vitro* kinetics of silicic acid release

**Figure 3.**
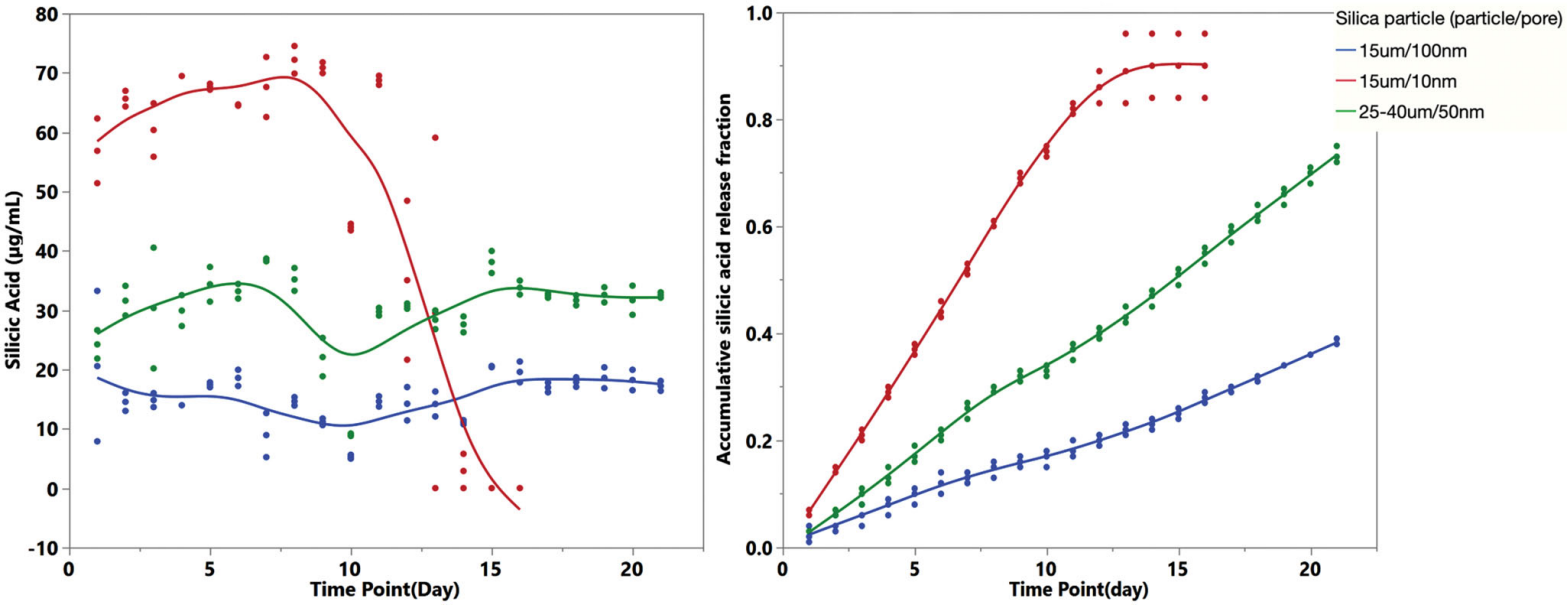
*In vitro* silicic acid dissolution kinetics from the sol-gel particles.

Figure 3 demonstrates the release profiles of silicic acid from the three formulations of particles. The average concentrations of silicic acid released per day were 54.35 µg/mL, 30.35 µg/mL and 15.38 µg/mL for the 15 µm/10 nm, 25–45 µm/10 nm and 15 µm/100 nm particles, respectively (Figure 3, left panel). The silicic acid dissolution rates from the particles were largely dependent on pore size. Within 3 weeks of dissolution, 90% had been dissolved from the 10 nm pore particles, while only 70% and 40% dissolution of the 50 nm and 100 nm pore particles occurred (Figure 3, right panel).

### *In vivo* ocular properties

The ocular safety and properties were evaluated in both rabbit and guinea pig eyes. Four formulations were evaluated in the rabbit eyes as shown in Table 2. The best formulation from the rabbit studies was evaluated in guinea pig eyes as a confirmatory second species.

**Table 2.** *In vivo* study layout and summary of ocular testing parameters.

Animal species	Sol-gel (particle/pore)	Targeted dose (mg/eye)	# of eyes	Inj. Vol (µL)	Mass balance (mg/eye)	AC cells (Fraction; Mean grade)	Vitreous haze around silica depot (Fraction; Mean grade)	Clinical retina finding	Histology
Rabbit	15 µm/**10 nm**	2	10	100	2.10	3*/10; 1.7	0/10; 0	Normal	Normal
Rabbit	15 µm/**10 nm**	8	4	100	8.16	0/4; 0	0/4; 0	Normal	Normal
Rabbit	15 µm /**30nm**	4	7	100	4.42	3/7; 1.1	2/7; 0.7	1/7; ON hyperemia	Confirms clinical findings
Rabbit	20–45 µm/**50 nm**	4	7	100	3.58	6/7; 1.6	5/7; 1.6	2/7; ON hyperemia	Confirms clinical findings
Rabbit	15 µm/**100 nm**	4	4	100	3.75	4/4; 3	4/4; 1.8	1/4; MR distortion	Confirms clinical findings
Guinea pig	15 µm/**10 nm**	0.64	10	20	0.64	3/10; 0.2	1/10; 0.3	1/10; RD	1/10; RD

ON: Optic Nerve; MR: Medullary Ray; RD: retinal detachment. Mean grade for AC cells and vitreous haze was derived from dividing the peak grade of all eyes by number of the eyes.

*Two animals with white masses in AC on day 3, one of them AC cleared by 2 weeks, the other never had cell reaction in AC. The 3rd animal had an AC reaction in both eyes that was clear by 2 weeks.

#### Rabbit eyes study

**Figure 4.**
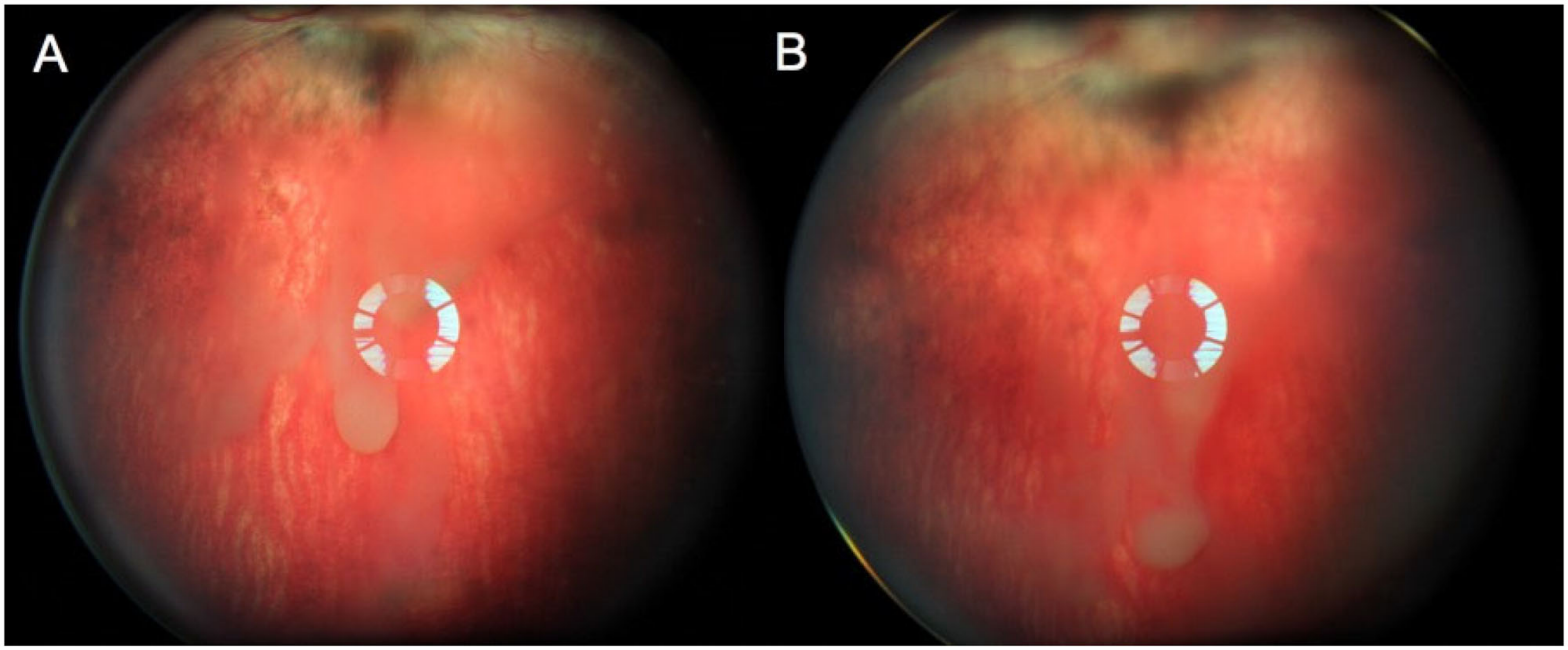
Rabbit fundus images. Panel (A) was taken 3 days after the intravitreal injection while panel (B) was taken 7 days after, showing the sol-gel particles settling down toward the inferior vitreous cavity over time.

Indirect ophthalmoscopy revealed that the sol-gel particles aggregated in the vitreous and settled down into the inferior vitreous cavity within the first week after intravitreal injection (Figure 4).

**Figure 5.**
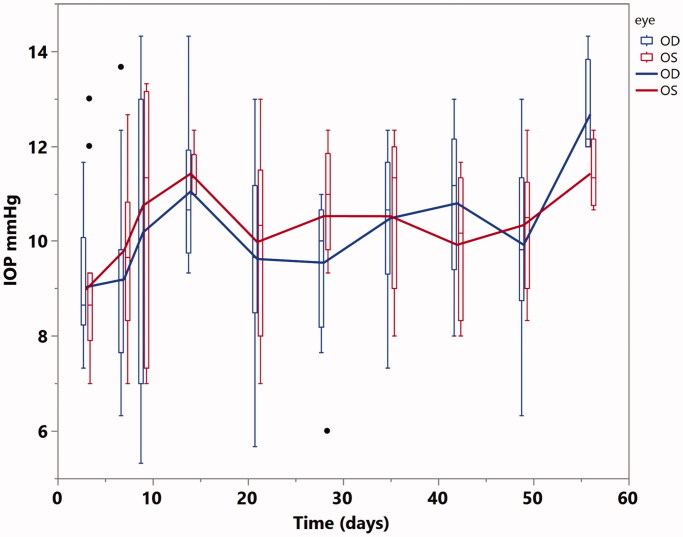
Intraocular pressure (IOP) of the eyes injected with the 15 µm/10 nm formulation (OD) versus the control eyes (OS) over the study course. There is no significant difference between OD and OS although IOP of both eyes increased over time.

Slit-lamp exams on day 3 revealed white masses in the aqueous humor in two rabbit eyes with the 2 mg dose of 15 µm/10 nm particles. The other 8 eyes with the 2 mg or 4 eyes with the 8 mg dose did not show any abnormalities. One of the two rabbits with a white mass was euthanized post-injection day 8 and histology showed lens damage and particles in the anterior chamber. The other eye with a white mass was closely monitored and the aqueous humor cleared by week 2. Formulations of with larger pores demonstrated variable localized vitreous cloudiness around the sol-gel particle depot and mild inflammatory cells in the aqueous humor (Table 2). The incidence of reaction around the depot was significantly increased in eyes with larger pored particles and there was a significant trend for higher incidence of vitreous reaction with larger pores (10 nm: 0%, 30 nm: 29%, 50 nm: 71%, 100 nm: 100%, *p* < .0001, Cochran Armitage Trend Test). Even taken 50 nm pored particle out (particle size is bigger (20–45 µm) than the other three pored particles (all 15 µm), the trend test is still significant (*p* < .0001) All rabbits with the 15 µm/10 nm formulation had normal intraocular pressure over the study course (Figure 5, least square means OD = 10 vs. OS = 10.2, *p* = .45; Time(days) *β* = 0.025, *p* = .0037) and normal ERGs at the end of the study when compared with the fellow control eyes (Dark adapted ERG mean b-wave: OS-OD = 5.8, Std Error = 3.7, *p* = .13; Light adapted ERG mean b-wave: OS-OD = –3.2, Std Error = 3.3, *p* = .35).

**Figure 6.**
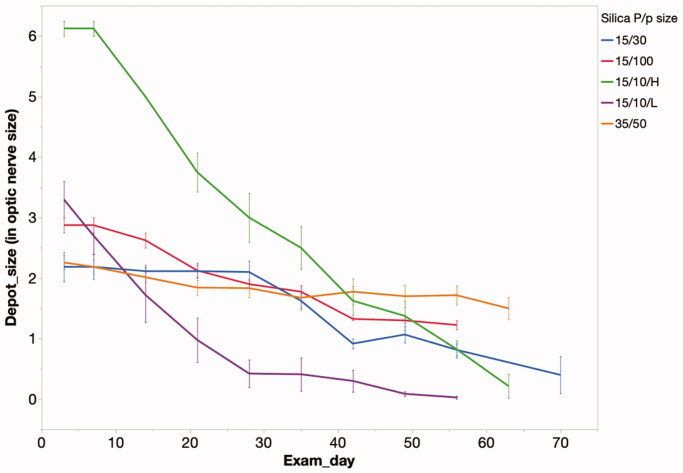
Particle depot size measured by optic nerve head area over time in the rabbit vitreous. The speed of degradation correlated with the release speed in vitro. 15 µm/10 nm low dose of pSi particles disappeared completely by week 8 while particles within the other groups were still visible. 15 µm/10 mm pSi particles showed the most rapid release, in both the high and low dose groups. P/p particle (particle/pore); 15/10/H = 15 µm/10 nm/high dose; 15/10/L = 15 µm/10 nm/low dose; 15/30 = 15 µm/30 nm; 35/50 = 35 µm/50 nm; 15/100 = 15 µm/100 nm.

The intravitreally injected sol-gel was monitored by indirect ophthalmoscopy over the study course. During these exams, the two-dimensional size of the depot was estimated by comparing it to the size of optic nerve head. The degradation profiles of the various sol-gel formulations are demonstrated in Figure 6.

The rabbit eye studies demonstrated that smaller pore (15 µm/10 nm) seemed to have good ocular compatibility while the larger pore formulations caused more reactions with varying degrees of aqueous humor flare and cells (1+ to 3+ Flare/Cells).

**Figure 7.**
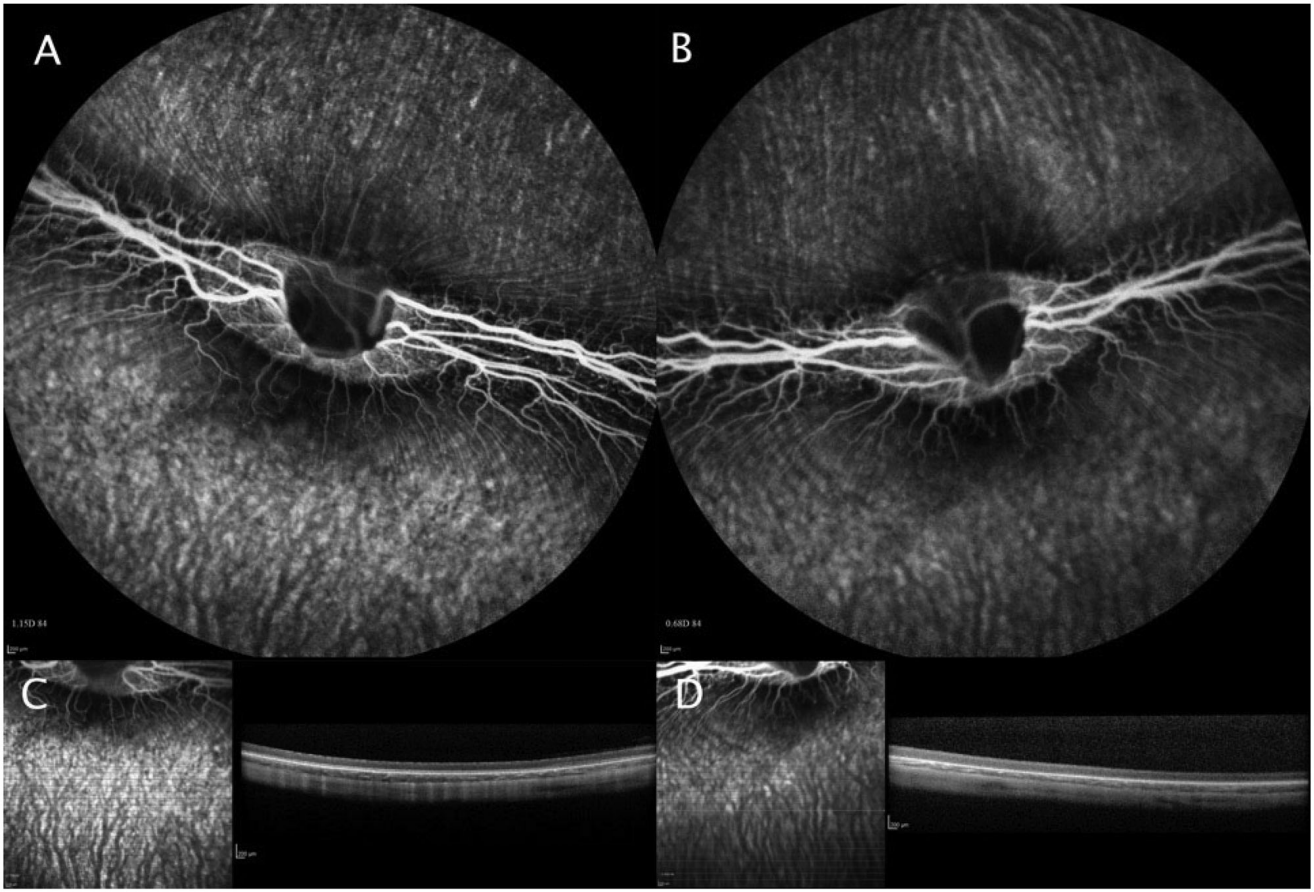
FA and OCT from a rabbit eye (A and C) injected with 15 µm/10 nm pSiO_2_ and its contralateral eye (B and D) injected with saline, images taken 8 weeks after the intravitreal injection. Both eyes demonstrated normal FA and OCT.

FA, OCT and ERG examinations were also performed for rabbits injected with 15 µm/10 nm pSi particles to verify the ocular safety of this particle formulation. No abnormal leakage was found in FA, while OCT examinations demonstrated normal retinal structure (Figure 7). Dark-adapted ERG b-wave amplitude of the injected eye versus the control eye was 149.60 ± 51.71 mV versus 165.00 ± 75.10 mV (*p* = .62) and the implicit time was 26.27 ± 2.14 ms versus 25.50 ± 3.18 ms (*p* = .33). Light-adapted ERG b-wave amplitude was 67.35 ± 29.02 mV versus 64.68 ± 27.88 mV and the implicit time was 18.91 ± 1.41 ms versus 19.45 ± 2.39 ms. For 30 Hz flicker ERG, the amplitude was 34.22 ± 13.61 mV versus 31.74 ± 17.68 mV and the time was 27.05 ± 0.58 ms versus 27.27 ± 1.93 ms.

#### Guinea pigs eye study

Ten guinea pigs were used as a second species for a confirmatory study of (15 µm/10 nm) formulation for 4 (5 guinea pigs) to 8 weeks (5 guinea pigs). Following intravitreal injection of the particles into the eyes of the guinea pigs, the particles were observed to aggregate in the vitreous similar to the particles’ behavior in rabbit vitreous noted above.

**Figure 8.**
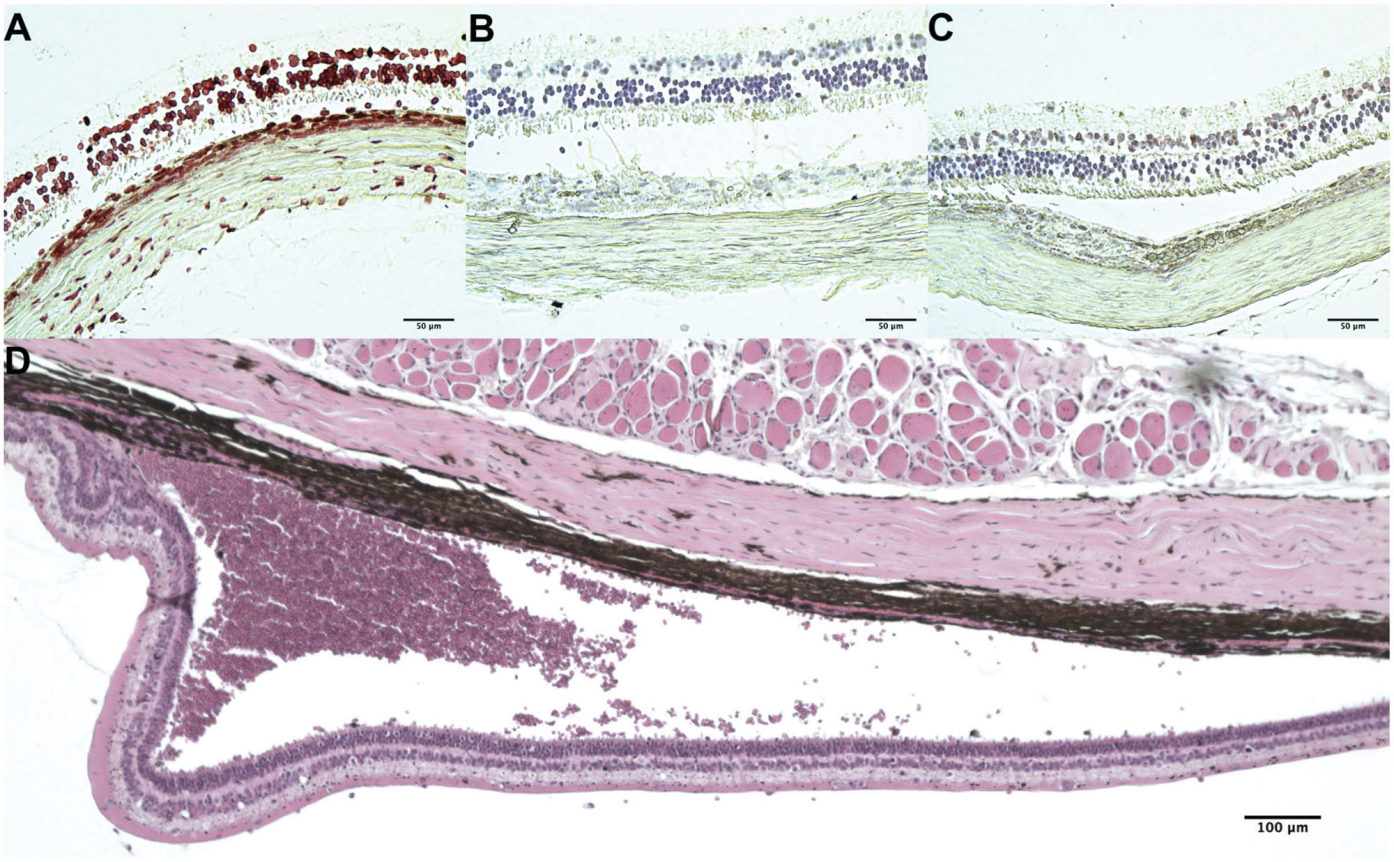
Guinea pig histology. (A) positive control for TUNEL (terminal deoxynucleotidyl transferase dUTP nick end labeling), (B) negative control for TUNEL, and (C) test eye TUNEL staining of guinea pig retina 8 weeks after the injection of sol-gel 15 µm/10 nm formulation. No detectable difference of apoptotic staining between the test eye retina and the negative control retina. (D) is an H&E stained slide from 4 weeks after injection, showing subretinal hemorrhage in the eye which showed aqueous cells and vitreous haze in clinical examination.

Clinically, 8 of the 10 guinea pigs had completely normal eyes. One guinea pig eye had slight aqueous humor cells (0.5 grade) due to vitreous hemorrhage from the injection procedure, which disappeared in the 4th week. The other guinea pig eye had aqueous cells and vitreous haze that lasted until the sacrifice 4 weeks after the intravitreal injection. All the other guinea pigs had FA and OCT before euthanasia and no abnormalities were noted. In addition, IOP was comparable with the contralateral eyes (right eye = 9.48 ± 3.0 mmHg versus left eyes = 8.81 ± 1.0 mmHg, *p* = .43, paired t-test). ERG exam prior to euthanasia demonstrated comparable amplitude and implicit time to the contralateral eyes. Dark-adapted ERG b-wave amplitude of injected eye versus the control eye was 57.94 ± 30.13 mV versus 50.84 ± 11.21 mV (*p* = 0.40) and the latency time was 17.10 ± 4.56 ms versus 19.00 ± 6.91 ms (*p* = .16). Light-adapted ERG b-wave amplitude was 48.13 ± 23.18 mV versus 51.10 ± 18.14 mV and the latency time was 22.30 ± 4.00 ms versus 21.30 ± 4.16 ms. For 30 Hz flicker ERG, the amplitude was 31.60 ± 5.46 mV versus 29.20 ± 2.49 mV and the latency time was 18.49 ± 10.54 ms versus 16.45 ± 7.90 ms. Histology investigation was focused on the 15 µm/10 nm pSi formulation, showing normal morphology and structures to confirm the normal clinical observations. Immunohistochemistry staining for apoptosis (TUNEL) did not reveal differences between the pSiO_2_ injected eyes and the control eyes (Figure 8(A–C)).

For the guinea pig with the observed vitreal haze, massive subretinal hemorrhage was revealed on the histology but very few inflammatory cells were seen in the vitreous cavity (Figure 8(D)).

## Discussion

Silicon is an element present in abundance on earth and in our body, though its biological roles are not very clear. Silicon dioxide, silica or glass, did not have much application in drug delivery until mesoporous silicon was developed. The silica particles portrayed in the current study is in the form of microparticles with nanometer scaled pores and fabricated in a sol-gel process. Silica or silicon dioxide, once in a living organism in the presence of biological fluids, degrades with resultant protonated silicic acid (H_4_SiO_4_) that has been identified in aqueous and vitreous humor after porous silicon or silicon dioxide particles were injected into the rabbit eye (Nieto et al., 2013). In contrast to porous silicon, no data is available for ocular safety of mesoporous sol-gel silica. Before the sol-gel silica particles were injected into the vitreous of living eyes, an in vitro cytotoxicity test was conducted using silicic acid disassociated from the same batch of sol-gel silica particles. This study was mimicking cells exposed to silicic acid degraded from intravitreal injected sol-gel silica particles. In the case of intravitreal injection, suspended silica particles release silicic acid into the vitreous and cleared from the eye through normal ocular fluid circulation and turnover. The current study demonstrated that 2.5 µg/mL silicic acid (26 µM) was not cytotoxic to EA.hy926 cells; however, 25 µg/mL (260 µM) was cytotoxic. This toxic concentration was much smaller than the cytotoxicity of silicic acid at 2 mM on mouse macrophage cell line (A640-BB-2 cells) and a fibroblast cell line (3T6 cells) reported by Tanaka et al (Tanaka et al., 1994). In the other studies, silicic acid concentration from 10^−4^ to 10^2^ mM was not toxic to B50 neuron cells (Mayne et al., 2000) but 204 µg/mL rendered survival of human osteoblast-like cell lines Saos-2 20% less than that of untreated cells (Duivenvoorden et al., 2008). The difference among these studies may stem from difference in cell lines, including their replication rate because rapidly proliferating cells tend to be more sensitive to the testing agents in the culture medium (Hou et al., 2011). In our study, we tested concentrations between the lower and upper concentrations (2.5 to 25 µg/mL) detected in aqueous and vitreous after injection of porous silicon into rabbit eyes (Nieto et al., 2013). In a living eye, due to constant ocular fluid moving dynamics silicic acid seldom maintains at high level and the silicic acid level at the most of time is below 25 µg/mL.

Although in vitro tests provide useful information in a well-controlled environment, they often cannot adequately recapitulate the complex responses in vivo. The current study in animal eyes demonstrated that particles with smaller pores (15 µm/10 nm) had a good ocular safety profile, although these particles had the fastest release rate of silicic acid and possibly higher resultant silicic acid concentration in the vitreous at the earlier time points. The ocular safety of this formulation (15 µm/10 nm) was tested in rabbit eyes and its safety confirmed in guinea pig eyes. In contrast, the other formulations (15 µm/30 nm, 20–45 µm/50 nm, and 15 µm/100 nm) all had higher rates of vitreous reaction around the depot. After excluding of larger particle size formulation (20–45 µm/50 nm), the remaining three formulations all had particle size of 15 µm but the pore sizes were still a significant factor for adverse reaction (vitreous haze around silica depot). Our experience suggests that vitreous haze around the depot, especially delayed vitreous haze, is more of an indication for ocular toxicity than mild acute inflammatory cells in the aqueous humor. The latter can derive from the injection procedure itself. The current study used micrometer scaled particles that have longer vitreous half-lives than their nanometer scaled counterparts. Longer vitreous half-life is an important parameter for intravitreal controlled drug delivery applications. In the current study, the mesoporous silica particles stayed suspended in the vitreous for a long time and the size of the particles seemed to not be critical in ocular safety, but pore size was a possible source of toxicity. We observed that with the same diameter of 15 µm, the particles with larger pores (30, 50, 100 nm) had more vitreous reaction around the particle depot. It is not clear why large pores are prone to induce vitreous reactions. In an in vitro study using porous silica nanoparticles, Tao et al. found that myeloid and lymphoid cells exposed to large pored silica showed toxicity by inhibition of cellular respiration (Tao et al., 2008). Another possibility is the format of degradation products of silica in the vitreous. It has been suggested based on some reports that polysilicic acids could lead to cytotoxicity by adsorbing or binding some enzymes or substrate proteins while monomeric silicic acid would not cause this and cause little to no cytotoxicity (He et al., 2009). How the large pores differ from small pores in respect to producing polysilicic acid is not well understood and cannot be concluded from the current study. However, surface physio-chemical features of the silica particles may play an important role in the ocular safety after the intravitreal injection. One interesting observation from our previous studies was that variable vitreous reactions are seen with unloaded porous silicon dioxide particles in rabbit vitreous but was never observed with therapeutic-loaded porous silicon dioxide particles (Chhablani et al., 2013; Hou et al., 2016). Another possibility for the observed ocular toxicity from the larger pored sol-gel silica particles could be that larger pored particles may more easily collapse into nano-sized particulates during degradation in the vitreous and nano-sized mesoporous silica has been reported to be more cytotoxic in vitro in human breast cancer cells and monkey kidney cells (He et al., 2009).

In summary, the mechanism by which mesoporous silica induces biological toxicity remains unclear (Tarn et al., 2013). Since different mesoporous silica are made under various conditions, reports of safety and toxicity varies from study to study (Hudson et al., 2008; Lin & Haynes, 2010; Yu et al., 2011). However, porous architecture plays a key role in toxicity (Bellocq et al., 2003; Bae et al., 2011; Lee et al., 2011). Most of the studies were performed in cultured cells or interfaced with solid animal tissues. Very few studies were conducted in the vitreous of a living eye (Cheng et al., 2008). The toxicity and safety may very likely be different in different biological environments. Eye vitreous is a special tissue that contains 99% water and roughly 1% collagen with minimal cells in it. How the dissoluted silicic acid interacts with collagen or vitreous cells as well as a mechanism of pore size effect needs to be further investigated. However, the current study demonstrated that sol-gel mesoporous silica microparticles with small pores (10 nm) are safe for intravitreal injection as drug delivery vehicles.
